# Italian Grape Ale Beers Obtained with Malvasia di Candia Aromatica Grape Variety: Evolution of Phenolic Compounds during Fermentation

**DOI:** 10.3390/foods12061196

**Published:** 2023-03-12

**Authors:** Giulia Leni, Elia Romanini, Terenzio Bertuzzi, Alessio Abate, Letizia Bresciani, Milena Lambri, Margherita Dall’Asta, Mario Gabrielli

**Affiliations:** 1 Department of Animal Science, Food and Nutrition (DIANA), Università Cattolica del Sacro Cuore, 29122 Piacenza, Italy; 2 Department for Sustainable Food Process (DiSTAS), Università Cattolica del Sacro Cuore, 29122 Piacenza, Italy; 3 Department of Food and Drug, University of Parma, 43124 Parma, Italy

**Keywords:** fermentation, flavonoid, fruit beer, grape must, grape pomace, HPLC-MS/MS, IGA beer, phenolic acids, (poly)phenol

## Abstract

Italian grape ale (IGA) beers have been categorized by the Beer Judge Certification Program as a sub-category of fruit beers in which grape, or grape must, is added during the brewing process to provide additional characteristics to the final beer. In the present work, IGA beers have been produced with must and pomace of the Malvasia di Candia Aromatica (MaCA) grape variety, which were added before fermentation at two different percentages (10% and 20%). The (poly)phenolic profile of IGA beers have been characterized with HPLC-MS/MS and compared to a golden ale control beer (produced in the same conditions without the addition of grape-derived ingredients). A series of sub-samples have been collected to monitor the (poly)phenol profile at time 0 and during the different phases of the fermentation process (1, 3, 5, 7 30, 65 days). Results demonstrated how the addition of pomace allowed to significantly enrich (*p* < 0.05) final beers in total (poly)phenols detected by MS, while must addition did not influence that amount if compared to the control sample. However, a PCA cluster analysis identified strong similarities among IGA beers and differentiated them to control beer samples. This study underlined how the addition of must and pomace from the MaCA grape variety improved the (poly)phenolic profile of beer from both a qualitative and quantitative point of view.

## 1. Introduction

Beer is the most consumed alcoholic beverage in the world [1]. In 2015, a new subcategory of beer has been proposed with the name Italian Grape Ale (IGA) [2], and commercial products are now available on the Italian market [3]. IGA beers differ from other beer products for the addition of grape or grape must in the formulation, thus representing a perfect linkage between the brewing and the winemaking sector. Furthermore, the use of surplus grapes and must, or winery waste (e.g., grape pomace), during the brewing process represents a valorizing opportunity to increase the sustainability of both strategic industrial assets. In particular, pilsner/pils or other pale base malts are used in combination with a variable amount of grape/grape must, which can range from 5% to 40% of the wort composition [3]. Furthermore, brewers can choose the brewing step for the grape addition, the type of high fermentation process (yeast from beer, wine or without yeast), making the wide range of these parameters an indication of the complexity of this style [4]. In 2021, De Francesco et al. investigated the physicochemical, volatile, and sensory properties of 22 commercial IGA beers available on the Italian market for the first time, identifying complex and heterogeneous profiles due to the processing differences [3]. However, some similar characteristics have been identified as well, such as high ethanol content, low bitterness and low pH [3]. Castro Marin et al. developed IGA beers obtained with the addition of an increased amount of Lambrusco red grape musts during the brewing process [5]. The addition of Lambrusco must influenced the beer color, which changed towards redder shades, and increased the total phenolic content and the volatile components, emphasizing some wine-derived sensory descriptors [6]. The addition of grape and grape must could represent a tool for tailoring organoleptic beer characteristics and increasing the concentration of bioactive compounds, such as carotenoids and (poly)phenols [6]. (Poly)phenols in beer have important physiological and technological properties, since they are directly involved in foam stability, colloidal formation, physico-chemical characteristics, and shelf-life [7]. Some of the most abundant (poly)phenols in beer are benzoic and cinnamic acid derivatives, flavonols, flavan-3-ols and proanthocyanidins, prenylflavonoids, and amino phenolic compounds, with phenolic acids and flavonoids, namely (epi)catechin, quercetin, myricetin and resveratrol, which are increased in fruit beers [6,8].

This work investigated the (poly)phenolic profile of a control Golden Ale beer produced in a pilot-plant and, for the first time, the effect on the bioactive compound profile caused by the addition, before alcoholic fermentation, of must and pomace of Malvasia di Candia Aromatica (MaCA), a typical Emilia-Romagna grape aromatical variety. Moreover, the (poly)phenol profile has been monitored during the different process stages, from fermentation to bottle storage, in order to investigate and evaluate the kinetic of (poly)phenols, thus emphasizing the differences among beers.

## 2. Materials and Methods

### 2.1. Reagent and Standards

All of the solvents, salts, acids and bases were of analytical grade and were purchased from Sigma-Aldrich-Merck (Darmstadt, Germany) or Carlo Erba (Milan, Italy). (Poly)phenol standards used for identification and quantification purposes with HPLC-MS/MS were as follows: protocatechuic acid, gallic acid, (+)-gallocatechin, (−)-epigallocatechin, caftaric acid, (+)-catechin, (−)-epicatechin, *trans*-coutaric acid, astringin, *trans*-fertaric acid, 2,6-dihydroxybenzoic acid, 4-hydroxybenzoic acid, chlorogenic acid, caffeic acid, vanillic acid, rutin, piceid, coumaric acid, sinapic acid, ferulic acid, luteolin, quercetin, apigenin, kaempferol, procyanidin B1 (Sigma-Aldrich-Merck, Darmstadt, Germany) and procyanidin B2 (Extrasynthese, Genay Cedex, France).

### 2.2. Experimental Design

**Figure 1:**
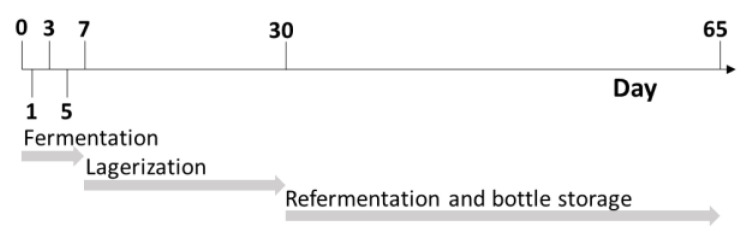
Schematic representation of sampling timepoints during different process stages.

Beermaking was carried out in a normal gravity process by means of a 12-hL plant (Spadoni, Orvieto, Italy). A Golden Ale wort was brewed with the addition of 250 kg of Pilsner Malt (Mouterij Dingemans, Stabroek, Belgium). The brewing steps were as follows: mashing-in was carried out at 60 °C with a water: malt ratio of 3:1 (750 L of water). The temperature of the mash was kept at 65 °C for 60 min (β-amylase rest). The mash out temperature was set at 78 °C. The temperature was raised 1 °C per minute. The mash was filtered by means of a thin bed membrane filter (Spadoni, Orvieto, Italy), and 550 L of water was used as sparging water. The spent grain water content was regulated until a value of 1.5° Plato was reached. The wort (1300 L) was boiled for 60 min with hops added under the form of type 90 pellets (0.3 g/L of cv. Hallertauer Magnum for 60 min and 0.80 g/L of cv. Styrian Goldings in the last five minutes). The wort was consequently whirlpooled for 20 min and the trub was completely removed. The wort (final volume: 1200 L) was filtered and immediately cooled to a temperature of 21 °C, with an initial extract content that was measured at 12° Plato. Extract content was determined at a temperature of 20 °C using a Densito 30PX manual oscillating densitometer (Mettler Toledo, Columbus, OH, USA). MaCA grapes were harvested at technological maturity in the Emilia-Romagna province of Piacenza. The grapes were destemmed and gently pressed with a hydraulic press at 0.8 bar for one minute (Model W40; Grifo Marchetti, Piadena, CR, Italy). Both the must and pomace were immediately stored at −20 °C. The must and pomace from the grape were defrosted at 4 °C overnight the same day as the beer wort production happened, which was on 28 October 2021. At the end of boiling, the grape must and pomace were pasteurized at 80 °C for 15 s and consequently blended with beer wort at two different percentage levels: 10% and 20%. A control beer was produced in the same condition without the addition of grape-derived ingredients. All the final blends were done in triplicate, for a total of 15 different batches of fermentation. All the worts were moved separately to 5 L plastic vats (Beer&Wine, Piacenza, Italy) and were inoculated with *Saccharomyces cerevisiae* (30 g/hL; SafAle US-05, Lesaffre, Marcq-en-Barœul, France). The fermentations were performed at 20 ± 1 °C and monitored daily by measuring the density (Densito 30PX manual oscillating densitometer, Mettler Toledo, Columbus, OH, USA) until the end of fermentation (day 7). Afterwards, the beers were racked off to separate solid parts from the liquids. Then, the beers were chilled to 4 °C for 23 days, and finally 4.8 g/L of sucrose was added with *Saccharomyces cerevisiae* (5 g/hL; SafAle F2, Lesaffre, Marcq-en-Barœul, France). The beers were bottled with screwcaps in 330 mL dark-glass bottles, and re-fermentation processes took place at 20 °C for 10 days and were monitored by checking the internal pressure using an afrometer (Oeno Italia, Brescia, Italy). Subsamples were collected at different timepoints as reported in Figure 1 after day 0, 1, 3, 5, 7, 30 and 65, and immediately stored at −20 °C.

### 2.3. (Poly)Phenol Extraction from Beers

Before analysis, each sample was gently shaken and degassed by ultra-sonication. Next, 100 µL of sample were mixed with 900 µL of water: acetonitrile (9:1 *v*/*v*) and transferred into a vial for the mass spectrometry analysis (MS).

### 2.4. (Poly)Phenol Analysis with HPLC-MS/MS

The system consisted of a Vanquish pump and autosampler, a TSQ Fortis triple-quadrupole mass spectrometer (Thermo-Fisher Scientific, San Jose, CA, USA). The separation was performed with a Betasil RP-18 column (5 µm particle size, 150 × 2.1 mm, Thermo-Fisher) with a gradient water: acetonitrile (both acidified with 0.2% of formic acid) of 90:10 maintained for 1 min, from 90:10 to 5:95 for 6 min, isocratic for 1 min, from 5:95 to 90:10 for 1 min and then isocratic for 10 min; the flow rate was 0.2 mL/min and the injection volume was 10 µL. (Poly)phenols were monitored with a spray voltage of +4000 V for positive transitions, or −3500 V for the negative ones; the ion transfer tube temperature was 270 °C, the vaporizer temperature was 200 °C, the sheath gas flow was of 35 units and the auxiliary gas flow was of 12 units. Detection of all the considered analytes was performed in the SRM modality (single reaction monitoring) by monitoring the characteristic transitions for each considered (poly)phenol (Table 1).

The performance of the method was evaluated by determining the limit of detection (LOD), the limit of quantification (LOQ), and the range of linearity (Table 1) for each compound. The linearity of calibration curves was very satisfactory, providing coefficient of determination values (R^2^) that were always above 0.995. Finally, the recovery percentages were assessed at two spike levels, and the results always provided a value above 95%. Individual compounds were quantified using a calibration curve of the corresponding standard compound.

### 2.5. Statistical Analysis

Data are expressed as mean ± standard deviation and are the mean of three different replicates. (Poly)phenol concentrations were Log transformed (base 10) and subjected to Principal Component Analysis (PCA) followed by k-means cluster analysis. The four most significative variables (from the PCA loading plot) were subjected to one-way analysis of variance (ANOVA) with Tukey’s Post Hoc test to determine the differences among sub-samples collected at different time points for each beer. Finally, a correlation analysis was performed on the entire dataset to identify the overall correlations between different features, and it was visualized with a heatmap correlation diagram. The elaboration was performed with MetaboAnalyst 5.0 (Available online at https://www.metaboanalyst.ca/; accessed on 15 June 2022) and SPSS version 21.0 (SPSS Inc., Chicago, IL, USA).

## 3. Results

**Figure 2:**
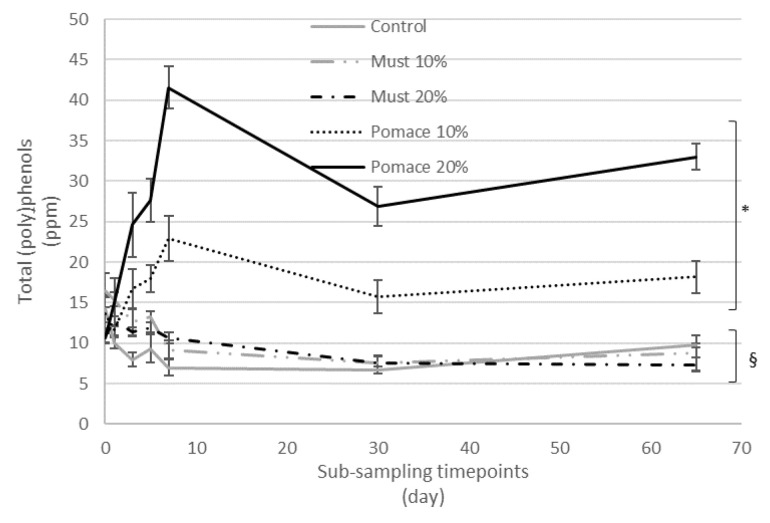
Kinetic of total (poly)phenols quantified by MS during the different timepoints from the beginning of fermentation to the final obtained beer (sub-samples collected at 0, 1, 3, 5, 7, 30 and 65 days). Results are expressed as ppm and are the mean of three separate analyses. * *p* < 0.05 indicates a significant increase in total (poly)phenols in the final beers, compared to the corresponding T0 value. § *p* < 0.05 indicates a significant decrease in total (poly)phenols in the final beers, compared to the corresponding T0 value.

The addition of must and pomace of MaCA at different percentages (10% and 20%) during the production of IGA beers influenced the amount of the total (poly)phenols quantified with MS in comparison to the control beer (Figure 2).

In particular, the total amount of (poly)phenols identified ranged between 7 ppm in beer enriched with 20% of must, to 33 ppm for beer enriched with 20% of pomace. To investigate the kinetic and fate of (poly)phenols during the whole brewing process, a series of sub-samples were collected from each beer at different time points and analysed for their (poly)phenol profile (Table S1 in the Supplementary Materials).

**Figure 3:**
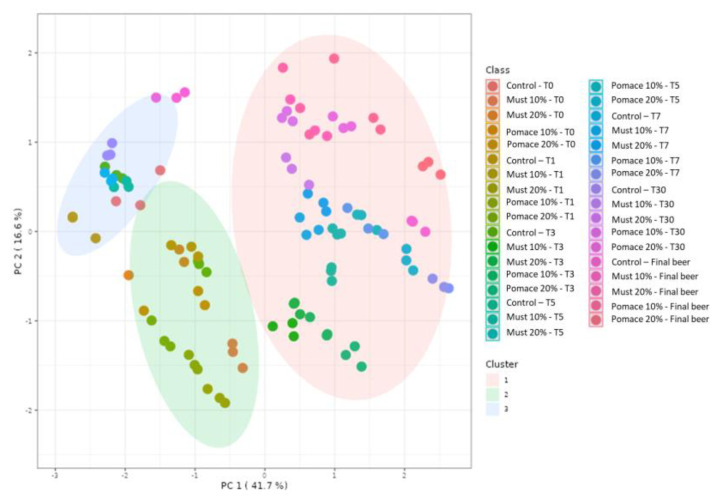
PCA score plot resulting from the unsupervised k-means clustering of the different (poly)phenols under investigation in the sub-samples collected during fermentation in the IGA and control beers, revealing three major groups according to the log-transformed (poly)phenol concentration.

The kinetic of total (poly)phenols identified by MS during the different steps from the first fermentation to the bottle storage is reported in Figure 2. The results demonstrated that the final beers (T65) obtained with the addition of pomace at both 10 and 20% were characterized by a significant increase of total (poly)phenols compared to the corresponding T0 samples. On the contrary, a significative decrease in total (poly)phenols was detected from the beginning of fermentation (T0) to the final beer in the control group and the must-enriched IGA samples. The entire dataset was then elaborated with a principal component analysis (PCA) (Figure 3), and 58% of the total variance was explained by the first two principal components (41.7 and 16.6%, respectively).

**Figure 4:**
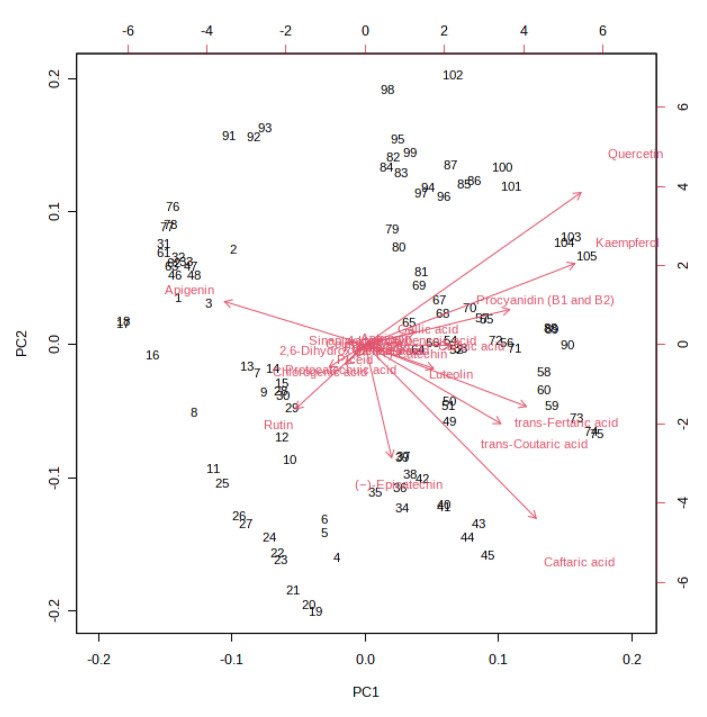
PCA loading plot resulting from the unsupervised k-means clustering of the different (poly)phenols under investigation in the different sub-samples collected during fermentation in IGA and control beers, revealing three major groups according to the log-transformed (poly)phenol concentration.

A K-means cluster analysis was performed in order to identify the potential presence of clusters and similarities between the whole dataset. The non-hierarchical cluster technique allowed for the identification of three different clusters (Figure 3): (i) 10% and 20% must and pomace IGA samples from the T3 to the final beer; (ii) 10% and 20% must and pomace IGA samples from the T0 to T1; and (iii) control samples from T0 to the final beer. The clusterization highlighted how the addition of 10 and 20% of must and pomace induced a systemic differentiation from the control group. In addition, among IGA samples, a further clusterisation has been made based on the sampling stage. A PCA loading plot (Figure 4) identified quercetin, kaempferol, caftaric acid and *trans*-fertaric acid as the most important contributors to distinguish the three clusters.

For these most significant (poly)phenols, *p* values of ANOVA were calculated to evaluate the presence of significative differences among their concentration at the different time points (Table 2).

**Figure 5:**
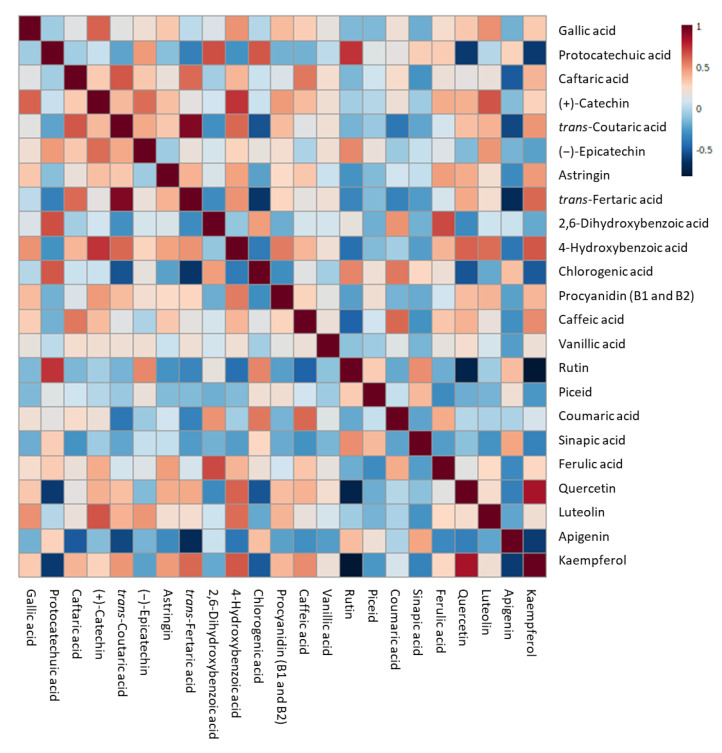
Heatmap representation of the correlation analysis between the log-transformed (poly)phenol concentration of different sub-samples of IGA and control beers. Each square indicates Pearson’s correlation coefficient value R^2^. Red and blue represent positive and negative correlation, respectively.

Quercetin, when detected, presented a range of concentration which varied from 49 ppb in the control sample at T0 to 2.8 ppm in the final beer produced with 20% of pomace. Except for the control samples, where the quercetin concentration remained lower than 1 ppm in the final beer, quercetin in IGA samples significantly increased by up to the 400% after 5 days from the starting of fermentation to the final beer. Caftaric acid and *trans*-fertaric acid were detected only in IGA samples. Caftaric acid concentration decreased in all samples during the monitored period, even if it became significant only for must-enriched samples after 7 days from the beginning of fermentation. On the contrary, the *trans*-fertaric acid concentration increased with the increasing of the fermentation period, reaching the maximum concentration at T5 in all IGA samples. Finally, kaempferol was detected in the control group only in the final beer, while in IGA samples it became detectable after 3 days of fermentation, then significantly increased in the final beers. To investigate the potential relationships between variables and the strength of these relationships, a correlation matrix was performed, and the correlation heatmap was reported in Figure 5.

Strong positive correlations (R^2^ > 0.7) were identified between protocatechuic acid and rutin, quercetin and kaempferol, *trans*-coutaric and *trans*-fertaric acid, and (+)-catechin and 4-hydroxybenzoic acid. On the contrary, strong negative correlations (R^2^ < −0.7) were determined with rutin compared to both quercetin and kaempferol.

## 4. Discussion

In 2015, IGA beers were proposed as a new sub-category of fruit beer obtained by the addition of grape or grape must to pilsner/pils or other pale base malts. In the present work, the MaCA grape variety was chosen for producing four different IGA beers, obtained by the addition of 10% and 20% of pomace or must before fermentation. The MS analysis allowed for the determination of the (poly)phenol profile of these IGA beers and its comparison to that of a control beer produced with the same method but without the addition of grape. IGA beers produced with pomace at both 10 and 20% presented a total amount of (poly)phenols quantified by MS, which was 86 and 239% higher than that of the control beer, respectively. On the contrary, the addition of must affected the total (poly)phenol content with respect to the control beer (on average 17% less), even if no significant differences were determined. The results underlined how the pomace and must of the MaCA grape variety differently influenced the amount of total (poly)phenols in beers detected by MS. These differences can be related to the different amount of total (poly)phenols in the starting material, with pomace which represented the raw product having a higher concentration of total (poly)phenols compared to must [9,10]. Gasiński et al. produced IGA beers with white grape pomace added after primary fermentation and determined a total (poly)phenol content 150% higher than that of a control lager beer [11]. Also, Veljovic et al. increased the content of total (poly)phenols by producing a lager beer using fermented grape must and wort [12]. In the present work, for the first time, the (poly)phenol profile of IGA beers produced with the MaCA grape variety has been determined. The addition of MaCA must and pomace provided characteristic (poly)phenols to the IGA samples. In particular, caftaric acid, *trans*-coutaric and *trans*-fertaric acid, cinnamate esters of tartaric acid, which do not characterize normal beers [13,14], were detected only in IGA samples, since they are the main hydroxycinnamic acids found in grapes [15]. On the contrary, the grape addition affected the content of gallic acid, protocatechuic acid, chlorogenic acid, rutin, and sinapic acid in the final IGA beers compared to the control sample, even if these differences became significant only for chlorogenic acid and rutin. Pomace and must presented a different (poly)phenol profile due to the uneven distribution of these compounds among the grape skin, seeds and pulp [16]. These differences, in some cases, were also reflected in the final beers. As an example, the pomace-enriched beers presented a higher concentration of caftaric acid, (+)-catechin, (−)-epicatechin, *trans*-coutaric acid, and caffeic acid if compared to the must-IGA beers, all (poly)phenols which are mostly located in the grape skin and seeds rather than the pulp [16,17]. However, these compounds in the IGA samples pre-fermentation (T0) presented concentrations which were comparable or even higher in the must enriched samples rather than the pomace ones, in contrast with their distribution in grapes. These results suggested that the hydrolytic activities of yeast occurred during fermentations could have contributed to the release of these compounds from pomace. Among the different (poly)phenols, (+)-catechin resulted as the most abundant in all final beers, with concentration ranging from 2 ppm in must-enriched beers to 11 ppm in the IGA beer with 20% of pomace. This bioactive compound, already known to be one of the most abundant beer flavonoids [14,18], together with (−)-epicatechin, is a precursor for the formation of proanthocyanidins, which provide structure and astringency to beer and are also correlated to haze formation [19]. Next to the final beers, a series of sub-samples have also been collected from each trial at different time-points and analysed as well for their (poly)phenol profile. Fermentations and lagerization had different effects on the content of total (poly)phenols in the control and IGA samples. In fact, in the control and must-enriched samples, a reduction in the total amount of (poly)phenols detected by MS was determined. This could probably be associated with the enzymatic decarboxylation of free hydroxycinnamic acids, catalysed by phenylacrylic acid and ferulic acid decarboxylase, or to their binding with proteins and polysaccharides [20]. By analysing the (poly)phenol profile of each sub-sample, it was demonstrated by PCA and cluster analysis that IGA samples, produced by both must and pomace, clustered together and separated from the control samples. In addition, if for control samples there was not any additional clusterisation based on the fermentation and lagerization stage, in IGA beers an additional cluster was demonstrated based on the sampling time points. These results demonstrated that, even if the total (poly)phenol content of pomace -enriched samples was statistically higher than that produced with must, the MaCA grape variety provided a similar (poly)phenolic profile to IGA beers produced with must and pomace. IGA appears to be a complex and heterogeneous beer style. Next to the grape variety and the brewing step in which the grape can be added, the yeast strain which was selected for the fermentation also plays a crucial role [21]. The results here reported underline how the fermentation performed by *Saccharomyces cerevisiae* strictly influenced the (poly)phenol profile of IGA beers from both a qualitative and a quantitative point of view. The ability of fermentation to improve the yield and to change the profile of phenolic compounds could be mainly associated with the release of bound phenolic compounds and to their metabolization in a large array of new compounds [22]. Unfortunately, the fate of each individual phenolic compound during the brewing process is somehow difficult to integrate, since they may undergo different pathways [23]. The IGA and control samples presented some similarity in the (poly)phenolic profile, as did the increased concentration of ferulic acid during fermentation. This behaviour has also been noticed by Coghe et al., who attributed this rise to the feruloyl esterase activity of *Saccharomyces cerevisiae* [24]. With regard to the differences among IGA and control beers, the negative correlation between rutin and quercetin has only been identified in IGA samples. This correlation can be ascribable to *Saccharomyces cerevisiae* glycosidase’s activity on rutin (structurally composed by quercetin bound to rutinose), with the subsequent release of free quercetin from the third day of fermentation. IGA beers were also characterised by the presence of a positive correlation between quercetin and kaempferol, probably due to the flavonol synthase activity exerted by *Saccharomyces cerevisiae* on the same dihydrokaempferol precursor [25].

In general, phenolic components of beer represent a great interest for brewers, since they directly affect the quality of beer, mainly in terms of its sensory characteristics. In addition, these plant-derived components may also exert biological activities within the human body. In fact, they are classified as non-nutrient molecules that are largely known for their antioxidant activity, but well described as compounds exerting a wide range of other biological functions related to the prevention of several non-communicable diseases [26,27]. However, it must be emphasized that the potential benefit associated with the higher amount of (poly)phenolic compounds present in IGA beers must be necessarily related to dietary guidelines on alcoholic beverages (including beer). In particular, it is strictly recommended to limit alcohol consumption as much as possible, not exceeding the low-risk dose (up to one alcoholic unit (corresponding to 1 beer (330 mL))/day for women and one alcoholic unit/day for men) to avoid the increased risk of developing chronic diseases, in particular cancer [28,29].

In the last decade, special beers produced with the addition of fruits, spices, and natural foods during the fermentation process have become very popular throughout the world, fulfilling the desire of consumers for new gustatory, olfactory, and visual stimuli [19]. Flavours and bioactive chemicals, such as (poly)phenols, are extracted from fruits added to beer during fermentation and maturation, contributing to boost the bioactive characteristics of beer. For this reason, fruit beers have been proposed to increase the content of bioactive compounds in beer, directly influencing its antioxidant potential [19]. However, besides their positive effect on preventing oxidation, they could negatively influence the colloidal and foam stability, directly affecting the shelf-life of beer. For this reason, further studies are needed in order to assess the shelf-life of IGA beers produced with pomace and must of the MaCA grape variety.

## Figures and Tables

**Table 1:** Characteristic transitions monitored for the targeted (poly)phenols and their limit of quantification (LOQ), limit of detection (LOD), and range of linearity.

Compound	Ionization Mode	Precursor Ion (m/z)	Product Ions (m/z)	Collision Energy (V)	Linear Range (ppm)	LOQ (ppm)	LOD (ppm)
Apigenin	Positive	271.1	91.1/118.9/152.9	+39/+30/+30	0.003–5.0	0.003	0.001
Astringin	Negative	451.3	200.9/243.1/405.1	–43/–25/–9	0.1–5.0	0.010	0.003
Caffeic acid	Positive	181.1	89.1/116.9/163.1	+29/+21/+9	0.1–5.0	0.100	0.030
Caftaric acid	Negative	311.1	135.1/149.1/178/9	–26/–8/–12	0.1–5.0	0.050	0.020
(+)-Catechin	Negative	335.2	203.0/245.1/289.0	–23/–18/–7	0.5–15.0	0.100	0.030
Chlorogenic acid	Positive	355.2	117.1/144.9/163.0	+24/+15/+9	0.1–5.0	0.050	0.015
Coumaric acid	Positive	165.1	91.1/119.0/147.0	+24/+18/+10	0.003–5.0	0.003	0.001
*trans*-Coutaric acid	Negative	295.2	113.1/119.1/163.0	–10/–23/–11	0.1–5.0	0.100	0.030
2,6-Dihydroxybenzoic acid	Negative	153.1	65.1/109.0/135.0	–19/–14/–13	0.04–15.0	0.010	0.003
(−)-Epicatechin	Negative	335.2	203.0/245.1/289.1	–23/–18/–7	0.04–15.0	0.010	0.003
(−)-Epigallocatechin	Negative	305.1	125.1/165.1/179.0	–19/–15/–13	1.0–5.0	1.000	0.300
*trans*-Fertaric acid	Negative	325.2	113.0/134.0/193.1	–10/–27/–12	0.1–5.0	0.050	0.015
Ferulic acid	Positive	195.1	89.1/144.9/177.1	+31/+16/+9	0.5–15.0	0.100	0.030
Gallic acid	Negative	169.1	79.04/71.1/124.9	–22/–17/–13	0.1–5.0	0.100	0.030
(+)-Gallocatechin	Negative	305.2	125.1/177.1/219.2	–20/–19/–15	1.0–5.0	1.000	0.300
4-Hydroxybenzoic acid	Negative	183.1	93.0/137.1/139.1	–19/–5/–5	0.1–5.0	0.100	0.030
Kaempferol	Positive	287.3	120.9/153.0/212.9	+30/+31/+28	0.1–5.0	0.050	0.020
Luteolin	Positive	287.3	135.1/153.0/241.1	+32/+32/+32	0.01–5.0	0.010	0.003
Piceid	Positive	391.2	106.9/135.0/229.1	+38/+26/+9	0.1–5.0	0.100	0.030
Procyanidin (B1 and B2)	Positive	579.3	291.1/409.1/427.2	+14/+18/+14	0.1–5.0	0.050	0.015
Protocatechuic acid	Negative	153.0	81.0/90.9/108.8	–23/–22/–13	0.05–15.0	0.050	0.015
Quercetin	Positive	303.1	136.9/152.9/	+30/+31/+28	0.01–5.0	0.010	0.003
Rutin	Positive	611.3	303.1/465.3	+20/+10	0.05–15.0	0.050	0.015
Sinapic acid	Positive	225.2	118.9/175.1/206.9	+20/+14/+8	0.1–5.0	0.100	0.030
Vanillic acid	Positive	169.1	64.9/93.1/125.0	+23/+14/+10	0.1–5.0	0.100	0.030

**Table 2:** Quercetin, caftaric acid, *trans*-fertaric acid, and kaempferol concentration in the different sub-samples collected from the starting of fermentation to the final beers.

Sample		Quercetin (ppm)			Caftaric Acid (ppm)			*Trans*-Fertaric Acid (ppm)			Kaempferol (ppm)		
**Control**	**T0**	0.05	±	0.01 ^b^		<LOD			<LOD			<LOD	
	**T1**		<LOD			<LOD			<LOD			<LOD	
	**T3**		<LOD			<LOD			<LOD			<LOD	
	**T5**		<LOD			<LOD			<LOD			<LOD	
	**T7**		<LOD			<LOD			<LOD			<LOD	
	**T30**		<LOD			<LOD			<LOD			<LOD	
	**Final beer**	0.07	±	0.01 ^c^		<LOD			<LOD		0.1	±	0.04 ^b^
**Must 10%**	**T0**		<LOD		2.07	±	0.95 ^bc^	0.4	±	0.02 ^a^		<LOD	
	**T1**		<LOD		3.09	±	0.59 ^c^	0.49	±	0.02 ^ab^		<LOD	
	**T3**		<LOD		3.07	±	1.02 ^c^	0.65	±	0.05 ^bc^	0.11	±	0.04 ^ab^
	**T5**	0.09	±	0.01 ^ab^	2.82	±	0.47 ^c^	0.72	±	0.02 ^c^	0.3	±	0.01 ^b^
	**T7**	0.15	±	0.02 ^b^	0.73	±	0.21 ^ab^	0.52	±	0.07 ^abc^	0.36	±	0.09 ^b^
	**T30**	0.16	±	0.02 ^b^	0.25	±	0.15 ^a^	0.6	±	0.07 ^abc^	0.31	±	0.06 ^b^
	**Final beer**	0.47	±	0.09 ^c^	0.32	±	0.08 ^a^	0.47	±	0.18 ^ab^	0.82	±	0.23 ^c^
**Must 20%**	**T0**		<LOD			<LOD		0.82	±	0.06 ^a^		<LOD	
	**T1**		<LOD		1.04	±	0.29 ^c^	0.97	±	0.01 ^ab^		<LOD	
	**T3**		<LOD		1.26	±	0.26 ^c^	1.25	±	0.02 ^bc^	0.18	±	0.01 ^b^
	**T5**	0.17	±	0.01 ^b^	0.61	±	0.07 ^b^	1.43	±	0.1 ^c^	0.32	±	0.03 ^bc^
	**T7**	0.18	±	0.02 ^b^	0.19	±	0.07 ^ab^	1.17	±	0.08 ^bc^	0.41	±	0.06 ^c^
	**T30**	0.24	±	0.04 ^b^		<LOD		1.31	±	0.21 ^c^	0.32	±	0.10 ^bc^
	**Final beer**	0.52	±	0.05 ^c^	0.15	±	0.03 ^a^	0.86	±	0.15 ^a^	0.4	±	0.08 ^c^
**Pomace 10%**	**T0**		<LOD		0.34	±	0.13 ^a^	0.13	±	0.004 ^a^		<LOD	
	**T1**		<LOD		0.3	±	0.12 ^a^	0.4	±	0.06 ^ab^		<LOD	
	**T3**		<LOD		1.37	±	0.22 ^b^	0.77	±	0.16 ^c^	0.22	±	0.03 ^b^
	**T5**	0.38	±	0.06 ^b^	0.34	±	0.11 ^a^	0.88	±	0.09 ^c^	0.32	±	0.03 ^bc^
	**T7**	0.43	±	0.08 ^b^	0.38	±	0.15 ^a^	0.6	±	0.15 ^bc^	0.29	±	0.06 ^bc^
	**T30**	0.58	±	0.09 ^b^		<LOD		0.76	±	0.14 ^c^	0.4	±	0.06 ^c^
	**Final beer**	2.41	±	0.32 ^c^	0.36	±	0.13 ^a^	0.58	±	0.08 ^bc^	1.01	±	0.13 ^d^
**Pomace 20%**	**T0**		<LOD			<LOD		0.4	±	0.08 ^a^		<LOD	
	**T1**		<LOD			<LOD		0.67	±	0.12 ^ab^		<LOD	
	**T3**		<LOD		2.45	±	0.28 ^c^	1.3	±	0.26 ^cd^	0.28	±	0.05 ^ab^
	**T5**	0.52	±	0.04 ^b^	1.21	±	0.32 ^b^	1.37	±	0.04 ^d^	0.37	±	0.07 ^b^
	**T7**	0.79	±	0.10 ^b^	3.03	±	0.8 ^c^	1.19	±	0.14 ^cd^	0.43	±	0.06 ^b^
	**T30**	0.81	±	0.05 ^b^	1.05	±	0.38 ^ab^	1.19	±	0.05 ^cd^	0.4	±	0.09 ^b^
	**Final beer**	2.84	±	0.31 ^c^	1.09	±	0.42 ^ab^	1.01	±	0.03 ^bc^	1.06	±	0.31 ^c^

Results are expressed as ppm and are the mean of three independent analyses. Statistical differences were determined for each phenolic compound between sub-samples of each beer. Different letters identified the presence of significative differences (ANOVA with Tukey’s Post Hoc test; *p* < 0.05). The bold should be maintained since they define the different type of samples characterized in the study, and differentiate them to the data collected.

## Data Availability

Data is contained within the article or supplementary material.

## Supplementary Materials

The following supporting information can be downloaded at: https://www.mdpi.com/article/10.3390/foods12061196/s1, Table S1: (Poly)phenol concentration in the different sub-samples collected from the starting of fermentation to the final beers.

## References

[1] Alcoholic Drinks—Worldwide | Statista Market Forecast. https://www.statista.com/outlook/cmo/alcoholic-drinks/worldwide.

[2] Beer Judge Certification Program—Promoting Beer Literacy, Recognizing Beer Tasting and Evaluation Skills. https://www.bjcp.org/.

[3] de Francesco G., Marconi O., Sileoni V., Perretti G. (2021). Barley Malt Wort and Grape Must Blending to Produce a New Kind of Fermented Beverage: A Physicochemical Composition and Sensory Survey of Commercial Products. J. Food Compos. Anal..

[4] Garavaglia C. (2020). The Emergence of Italian Craft Breweries and the Development of Their Local Identity. Geogr. Beer: Cult. Econ..

[5] Castro Marin A., Baris F., Romanini E., Lambri M., Montevecchi G., Chinnici F. (2021). Physico-Chemical and Sensory Characterization of a Fruit Beer Obtained with the Addition of Cv. Lambrusco Grapes Must. Beverages.

[6] Nardini M., Garaguso I. (2020). Characterization of Bioactive Compounds and Antioxidant Activity of Fruit Beers. Food Chem..

[7] Fumi M.D., Galli R., Lambri M., Donadini G., de Faveri D.M. (2011). Effect of Full-Scale Brewing Process on Polyphenols in Italian All-Malt and Maize Adjunct Lager Beers. J. Food Compos. Anal..

[8] Baigts-Allende D.K., Pérez-Alva A., Ramírez-Rodrigues M.A., Palacios A., Ramírez-Rodrigues M.M. (2021). A Comparative Study of Polyphenolic and Amino Acid Profiles of Commercial Fruit Beers. J. Food Compos. Anal..

[9] Negro C., Tommasi L., Miceli A. (2003). Phenolic Compounds and Antioxidant Activity from Red Grape Marc Extracts. Bioresour. Technol..

[10] Lachman J., Šulc M., Faitová K., Pivec V. (2009). Major Factors Influencing Antioxidant Contents and Antioxidant Activity in Grapes and Wines. Int. J. Wine Res..

[11] Gasiński A., Kawa-Rygielska J., Mikulski D., Kłosowski G., Głowacki A. (2022). Application of White Grape Pomace in the Brewing Technology and Its Impact on the Concentration of Esters and Alcohols, Physicochemical Parameteres and Antioxidative Properties of the Beer. Food Chem..

[12] Veljovic M., Djordjevic R., Leskosek-Cukalovic I., Lakic N., Despotovic S., Pecic S., Nedovic V. (2010). The Possibility of Producing a Special Type of Beer Made from Wort with the Addition of Grape Must. J. Inst. Brew..

[13] Quifer-Rada P., Vallverdú-Queralt A., Martínez-Huélamo M., Chiva-Blanch G., Jáuregui O., Estruch R., Lamuela-Raventós R. (2015). A Comprehensive Characterisation of Beer Polyphenols by High Resolution Mass Spectrometry (LC–ESI-LTQ-Orbitrap-MS). Food Chem..

[14] Neveu V., Perez-Jiménez J., Vos F., Crespy V., du Chaffaut L., Mennen L., Knox C., Eisner R., Cruz J., Wishart D. (2010). Phenol-Explorer: An Online Comprehensive Database on Polyphenol Contents in Foods. Database.

[15] Garrido J., Borges F. (2013). Wine and Grape Polyphenols—A Chemical Perspective. Food Res. Int..

[16] Qi M., Luo Z., Wu B., Wang L., Yang M., Zhang X., Lin X., Xu Y., Li X., Li L. (2022). Spatial Distribution and Time-Course of Polyphenol Accumulation in Grape Berry (Vitis Labruscana Cv. ‘Kyoho’). J. Food Compos. Anal..

[17] LI F.X., LI F.H., YANG Y.X., YIN R., MING J. (2019). Comparison of Phenolic Profiles and Antioxidant Activities in Skins and Pulps of Eleven Grape Cultivars (*Vitis vinifera* L.). J. Integr. Agric..

[18] Arranz S., Chiva-Blanch G., Valderas-Martínez P., Medina-Remón A., Lamuela-Raventós R.M., Estruch R. (2012). Wine, Beer, Alcohol and Polyphenols on Cardiovascular Disease and Cancer. Nutrients.

[19] Radonjić S., Maraš V., Raičević J., Košmerl T. (2020). Wine or Beer? Comparison, Changes and Improvement of Polyphenolic Compounds during Technological Phases. Molecules.

[20] Carvalho D.O., Guido L.F. (2022). A Review on the Fate of Phenolic Compounds during Malting and Brewing: Technological Strategies and Beer Styles. Food Chem..

[21] (PDF) The Influence of Raw Materials and Fermentation Conditions on the Polyphenol Content of Grape Beer. https://www.researchgate.net/publication/279481687_The_influence_of_raw_materials_and_fermentation_conditions_on_the_polyphenol_content_of_grape_beer.

[22] Thai Huynh N., van Camp J., Smagghe G., Raes K., Thanh Ward T., Phu District T., Chi Minh City H. (2014). Improved Release and Metabolism of Flavonoids by Steered Fermentation Processes: A Review. Int. J. Mol. Sci..

[23] Ambra R., Pastore G., Lucchetti S. (2021). The Role of Bioactive Phenolic Compounds on the Impact of Beer on Health. Molecules.

[24] Coghe S., Benoot K., Delvaux F., Vanderhaegen B., Delvaux F.R. (2004). Ferulic Acid Release and 4-Vinylguaiacol Formation during Brewing and Fermentation: Indications for Feruloyl Esterase Activity in Saccharomyces Cerevisiae. J. Agric. Food Chem..

[25] Chrzanowski G. (2020). Saccharomyces Cerevisiae—An Interesting Producer of Bioactive Plant Polyphenolic Metabolites. Int. J. Mol. Sci..

[26] García-Conesa M.T., Larrosa M. (2020). Polyphenol-Rich Foods for Human Health and Disease. Nutrients.

[27] Fraga C.G., Croft K.D., Kennedy D.O., Tomás-Barberán F.A. (2019). The Effects of Polyphenols and Other Bioactives on Human Health. Food Funct..

[28] Alcoholic Drinks and Cancer Risk—WCRF International. https://www.wcrf.org/diet-activity-and-cancer/risk-factors/alcoholic-drinks-and-cancer-risk/.

[29] Linee Guida per Una Sana Alimentazione 2018—Linee Guida per Una Sana Alimentazione 2018—Food and Nutrition—CREA. https://www.crea.gov.it/en/web/alimenti-e-nutrizione/-/linee-guida-per-una-sana-alimentazione-2018.

